# Seasonal Variation in Nocturnal Roost Timing and Diurnal Movement in Endangered Crested Ibis (*Nipponia nippon*): An Adaptation Strategy to Environmental Changes

**DOI:** 10.3390/biology14111496

**Published:** 2025-10-26

**Authors:** Wei Li, Dongping Liu, Yuhe Liao, Ke He, Chao Wang

**Affiliations:** 1 Key Laboratory of Biodiversity Conservation of National Forestry and Grassland Administration, Ecology and Nature Conservation Institute, Chinese Academy of Forestry, Beijing 100091, China; livi54@163.com (W.L.);; 2 College of Animal Science and Technology, College of Veterinary Medicine, Zhejiang Agriculture and Forestry University, Hangzhou 311300, China; 3 Shaanxi Hanzhong Crested Ibis National Nature Reserve, Hanzhong 723300, China

**Keywords:** *Nipponia nippon*, daylight utilization, activity level, GPS tracking

## Abstract

**Simple Summary:**

We used long-term observation data on roost timing and the GPS tracking data to analyze the adaptation strategies of the endangered Crested Ibis (*Nipponia nippon*) to environmental changes. Our findings reveal that Crested Ibises adopt three seasonal behaviors: extending the dawn and dusk activity window, increasing daylight utilization, and reducing daily movement distance and activity levels, so as to cope with the challenges in winter.

**Abstract:**

The living environment of birds exhibits seasonal variations, and winter cold and food shortages are key limiting factors influencing the survival rate of many bird species. However, most previous studies have focused on dynamic habitat changes, with relatively few investigating how changes in birds’ behavioral rhythms and ecological adaptability respond to seasonal fluctuations in the environment. The Crested Ibis (*Nipponia nippon*) is an endangered species, with winter food shortage being a critical factor constraining its population growth. Through 211 days of monitoring on the communal roosting behavior and GPS tracking of 19 individuals, this study aimed to clarify seasonal variations in their time allocation and activity levels, and reveal how Crested Ibises respond to environmental changes. During the wintering period, Crested Ibises departed nocturnal roosts earlier relative to sunrise and returned later relative to sunset, thereby utilizing more dawn and dusk time for foraging and increasing daylight utilization. GPS tracking data showed that both daily movement distance and activity levels of Crested Ibises during the wintering period were significantly lower than in other seasons—a pattern likely representing an adaptive strategy to cope with limited food resources, as it serves to minimize energy expenditure and enhance survival rates. Thus, these findings indicate that Crested Ibises adapt to wintering environmental changes through three seasonal behaviors: extending the dawn and dusk activity window, increasing daylight utilization, and reducing daily movement distance and activity levels.

## 1. Introduction

Seasonal environment changes profoundly impact birds, deeply shaping their life history traits [1]. For migratory birds, large-scale geographic migration is a typical adaptative mechanism to avoid unfavorable environmental conditions and seek habitats with abundant food resources [2,3]. In contrast, resident birds face different challenges and must adapt to regional environmental changes in their habitats.

Resident birds employ various mechanisms to cope with seasonal changes in climate and food resources to maintain survival and reproduction. For example, they often gather in groups and roost communally to resist cold and improve foraging efficiency [4,5]. Such collective behaviors are considered to provide a certain amount of heat, reduce the individuals’ exposure to harsh weather, and enhance safety during roosting and foraging [6,7,8]. Meanwhile, adjusting diurnal behavioral rhythms represents another mechanism. As daylight hours shorten in winter, resident birds tend to forage as much as possible to maximize food intake [9]. In response to reduced food abundance during wintering season, birds adjust their daily activity allocation and use available resources to cope with the seasonal environmental changes [10,11]. In addition, reducing activity levels and energy consumption during colder seasons is a key strategy to address food scarcity [12]. This behavioral adaptation helps birds reduce energy expenditure, ensuring they have sufficient resources to survive harsh seasonal periods, especially when food availability is minimal [13,14].

The Crested Ibis (*Nipponia nippon*) is a globally endangered species [15] and a National First-class Protected Animal in China. In history, this species was widely distributed in China, Russia, Japan and Korean Peninsula, with a migratory population in Northeast Asia and a resident population in Central China. Due to habitat loss, illegal hunting, and other human disturbances similar to those affecting the Northern Bald Ibis (*Geronticus eremita*) [16,17], its population declined dramatically in the first half of the 20th century. This led to the extinction of the eastern migratory population, leaving only an extremely small resident population remaining in Shaanxi Province, China [16]. Despite partial recovery due to conservation efforts, the species still faces multiple survival challenges, among which seasonal variation in food availability significantly impacts individual survival and population growth [18,19]. Crested Ibis primarily feeds on grasshoppers, loaches and other small fishes in winter-flooded rice fields, riverbanks and wetlands along reservoir banks [16,18]. Field observations indicated that starvation caused by winter food shortages is a major cause of increased mortality in rescued individuals [20]. Notably, the extensive temporal overlap between moult and breeding seasons in this Crested Ibises may represent a life-history strategy to avoid periods of extreme food scarcity in winter, thereby optimizing energy allocation [21]. These findings demonstrated that the Crested Ibis has developed multiple adaptive strategies to cope with environmental stress, including both physiological and behavioral adjustments.

As a communal roosting species, the Crested Ibis exhibits significant seasonal variations in communal roosting behavior. During the breeding season (February–June), paired Crested Ibises tend to roost in or in close proximity to their nesting trees. Following the breeding season, the species shifts into a wandering season (July–October), during which individuals gradually disperse to their wintering grounds. A key behavioral feature of this wandering phase is the formation of aggregations, with communal nocturnal roosting becoming the dominant roosting strategy during this period. Throughout the entire wandering season and subsequent wintering season (November–January of the following year), the dynamic formation and reorganization of these aggregations drive corresponding shifts in the spatial distribution and composition of their nocturnal roost sites [13,18]. Specifically, the timing, duration, and spatial distribution of roosting activities show distinct seasonal patterns, likely reflecting the species’ response to fluctuating resource availability and climatic factors. We hypothesize that these seasonal adjustments in roosting time may serve as an important behavioral adaptation to environmental pressures. However, the specific mechanisms by which communal roosting behavior mitigates winter survival pressures remain unclear.

Numerous studies have addressed winter food shortages through the restoration of foraging habitats, but few have explored how Crested Ibises cope with seasonal food changes as the main limiting factor [19,22,23]. Some findings indicate that Crested Ibises exhibit behavioral plasticity and ecology adaptability in allocating the timing of daily activities between roosting and foraging based on weather conditions, but this has been insufficiently studied. Based on this, we hypothesize that the allocation of foraging time has a significant impact on the wintering season of these birds, and their time allocation pattern—especially in winter—can be identified through roosting time. In this study, we investigated the roost timing of Crested Ibis populations over 6 years and GPS-tracked 19 individuals to reveal seasonal variation in nocturnal roost timing and daytime activity, so as to better understand the adaptation strategy under environmental changes.

## 2. Materials and Methods

### 2.1. Roosting Counts

**Figure 1:**
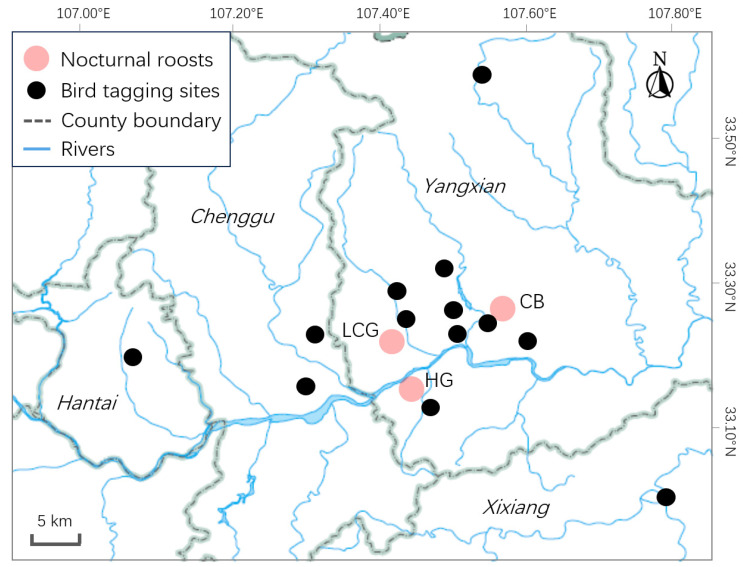
Location of the three nocturnal roosts and GPS tagging sites of Crested Ibis in the study.

To address the seasonal roost timing, roosting counts were conducted in non-breeding season at three nocturnal roosts of wild Crested Ibis in Yangxian County, Shaanxi Province, China (Figure 1). The first roost Caoba (CB, 33.2533° N, 107.5543° E) was a woodland dominated by *Cyclobalanopsis glauca* with an area of approximately 1.5 ha. The second roost Leicaogou (LCG, 33.2158° N, 107.4212° E) consisted of several *Ailanthus altissima* trees. The third roost Huoguang (HG, 33.1673° N, 107.4421° E) was a small woodland mainly composed of *Cyclobalanopsis glauca* and *Albizia julibrissin*. The three roosts were located in relatively close proximity, with distances ranging from 6 to 14 km apart, forming a cluster within the core habitat area of the Crested Ibis in Yangxian County. Individuals frequently moved between these roosting sites and shared part of the foraging habitats.

Crested Ibises usually arrived at roosts in flocks between 16:30 and 19:30 and departed roosts between 5:30 and 7:30, depending on seasons, daylight, and weather. Observers arrived at the roosts approximately 30 min before the expected arrival and departure times of birds. High ground around the roosts with good visibility were selected to count all birds flying in or out. After arriving at the roosting site in the afternoon, we first checked whether there were any Crested Ibises already present. If there were, we recorded them as an arriving flock at the current time. Roosting counts were conducted by a team of two observers: one counted the birds in each arriving or departing flock, while the other recorded the time, number of birds, and light intensity. We collected light intensity in the wild on sunny days, and it was measured using an illuminometer (Model ST-80C, Photoelectric Instrument Factory of Beijing Normal University, Beijing, China) placed in an open area. In total, 211 days of arrival counts and 170 days of departure counts were recorded. During this period, the number of Crested Ibises roosting at each site varied typically from 50 to 200, depending on seasons.

### 2.2. GPS-GSM Transmitter Attachment

From 2014 to 2022, 19 wild Crested Ibises were tracked using GPS-GSM transmitters (HQBN2525 and HQBN3527, Hunan Global Messenger Technology Co., Ltd., Changsha, China; Figure 1, Table 1). Of which, the 11 nestlings were captured from their nests at 25–30 days of age, using hand net by experienced personnel from the local National Nature Reserve. At this age, nestlings are nearing adult weight but are not yet capable of flight, facilitating safe capture and handling [24]. The rest 8 adults or sub-adults were all rescued individuals. Upon capture, the Crested Ibises were placed in individual cloth bags, and were recorded the mass, wing length and tarsus length. Then, the GPS tag was fitted using a Teflon ribbon back harness (Hunan Global Messenger Technology Co., Ltd., Changsha, China). Each tag was solar-powered, and the total weight of the harness accounted for 1.6% to 1.9% of the individual’s body weight, with repositioning every hour [25]. For some individuals, we collected a fresh abdominal feather, utilized tissue samples from the feather pulp, and identified the sex of Crested Ibis by the PCR-based sex identification technology [26]. After processing, nestlings were returned to their original nests, while rescued adults were released at suitable nearby habitats. The tracking days averaged 879 ± 596 days (ranged 252~2309 days). Regarding the reasons for tracking failures: among the 19 tagged birds, 3 were found dead, 3 had tags that ran out of power, 3 experienced tag malfunctions, and the causes for the remaining 10 were unknown (Table 1).

### 2.3. Roost Timing and Activity Time

Roost timing was analyzed using data from the communal roosting counts. The median time of arrival or departure (i.e., the time when 50% of the total Crested Ibis population have arrived at or departed from the communal nocturnal roosts) was used to represent the arrival and departure dynamics of communally roosting Crested Ibis [27]. Median light intensity was defined as the light intensity at the median arrival or departure time. Roosting arrival/departure duration was defined as the time between the arrival/departure of the first bird and the last bird at/from the roost. Seasonal dynamic in the Crested Ibis arrival and departure times relative to sunset and sunrise (obtained from China Meteorological Database via http://data.cma.cn/) were visualized using the R package “ggplot2” version 3.4.4, and the linear regression lines with the specification of “method = lm” were plotted to fit the trends [28]. Subsequently, to quantify the relationship between roost timing and population size, we fitted separate Linear Mixed Models (LMMs) using the “lme4” package version 1.1-35.2. In these models, roosting timing (arrival or departure) served as the dependent variable, population size was included as a fixed effect, and both “Date” and “Roost Site” were incorporated as random intercepts to control for the non-independence of repeated measurements. Finally, depending on the fitting smoothness, the influence of light intensity on roost timing was used “method = loess” to regress the seasonal trends. This method generates a smooth curve via local polynomial regression (bandwidth parameter span = 0.75) and reduces outlier influence through weighting functions [29].

Activity time is calculated by the interval between the departure and arrival time of the same roosts. To quantify adaptive behavioral adjustments of birds to the changes in daylight duration, the Daylight Utilization Ratio (DUR) was defined as:

DUR = Activity Time/Daylight Duration

DUR was compared between the wandering and wintering seasons using the Wilcoxon rank sum tests. The complex nonlinear relationship between DUR and seasonal daylength was visually explored using a smoothing spline (method = “gam”) within the “ggplot2” package to illustrate the trend [30]. The smoothing parameter was selected via restricted maximum likelihood (REML), with 95% confidence intervals representing uncertainty estimates. For trend in departure and arrival durations across months, local weighted regression (LOESS) was used for non-parametric fitting [29]. A two-way analysis of variance ANOVA was conducted to examine the effects of month, group (arrival/departure), and their interaction on duration. Assumptions of normality (Shapiro–Wilk test) and homogeneity of variances (Levene’s test) were verified prior to analysis.

### 2.4. Daily Movement Distance and Activity Level

The movement patterns of Crested Ibis were analyzed based exclusively on data obtained from the GPS-tracked individuals. Daily locations were analyzed using filtered data with speeds less than 5 km/h. Daily movement distance was calculated based on chronologically ordered daily locations. Only dates with at least fourteen fixes were included in the analysis. The average daily movement distance was computed for three periods: breeding season (February–June), wandering season (July–October, when the birds disperse from breeding grounds to wintering grounds) and wintering season (November–January) [18].

In addition, the activity level of individuals was measured using Overall Dynamic Body Acceleration (ODBA), a reliable proxy for energy expenditure derived from the tri-axial accelerometers within the transmitters [31]. The accelerometer sampled data every 10 min, with each sample consisting of an 8-s recording at 10 Hz on all three axes. ODBA was calculated for each sample by first subtracting a 1-s running average from the raw acceleration data of each axis to isolate the dynamic component, and then summing the absolute values of these dynamic accelerations across all three axes. The mean daily ODBA value was then computed for each individual and used as the metric for activity level in all subsequent analyses. Since fledglings remain highly dependent on adults during the post-fledging period [24], therefore we excluded the data of first-year juvenile from analysis.

For daily movement distance and activity level analysis, a linear mixed effects (LMM) model was used to evaluate the impact of different seasons, with individual differences treated as random effects. Post hoc pairwise comparisons between seasons were performed using estimated marginal means (EMMs) with Tukey’s adjustment for multiple testing (“emmeans” package version 1.11.0). Significance was assessed at α = 0.05, and *p*-values were adjusted to control the family-wise error rate. The Wilcoxon rank-sum test was used to determine whether there were significant differences in daily movement distance and activity level across seasons.

All data were analyzed with R statistical software version 4.3.1 [32]. The “car” package version 0.7.2 [33] was used for one-way repeated measures ANOVA, and the “rstatix” package version 0.6.0 [34] for two-way analysis of variance. All figures were created using the “ggplot” package and “ggboxplot” package version 0.4.0 [28,35].

## 3. Result

### 3.1. Seasonal Variation in Roost Timing

**Figure 2:**
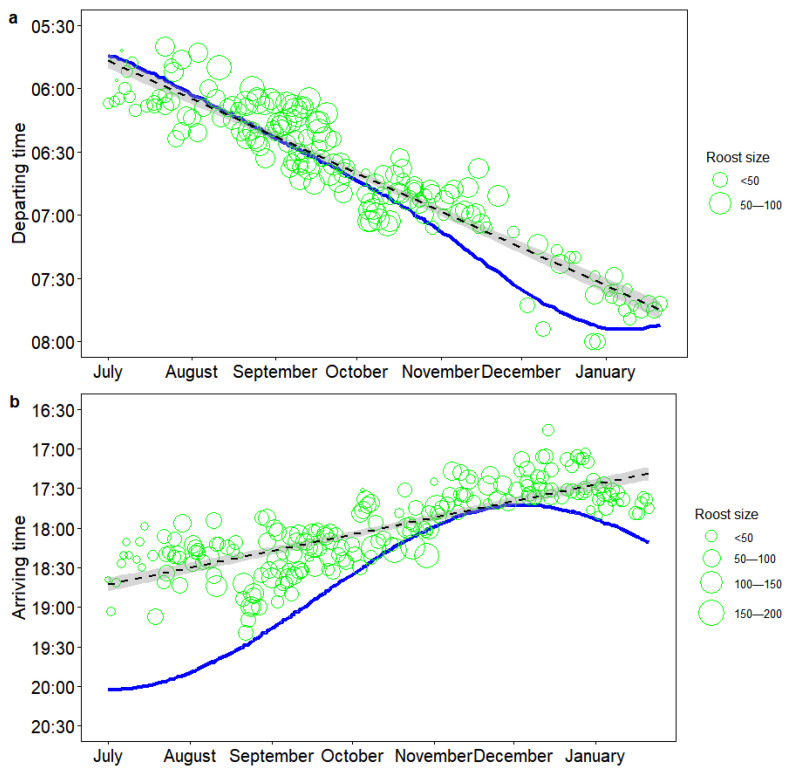
Seasonal changes in median departure time relative to sunrise ((**a**), *n* = 170 observation days) and median arrival time relative to sunset ((**b**), *n* = 211 observation days). The blue line represents sunrise/sunset times, green circles represent departure/arrival times, black dashed lines are fitted linear regression lines, and gray areas represent standard errors.

Crested Ibises’ departure and arrival times to the communal roosts always coincided with sunrise and sunset. However, from July to the following January, the departure times were generally getting earlier relative to sunrise (from 8.9 ± 11.4 min after sunrise in July to 13.3 ± 5.6 min before sunrise in January). As winter approached the solstice, Crested Ibises were observed leaving roosts approximately 30 min before dawn (Figure 2a). On the other hand, the arrival times were getting later relative to sunset (from 92.8 ± 17.0 min before sunset in July to 21.6 ± 6.9 min before sunset in January; Figure 2b). The analysis revealed that as the population size at the roost increased, the median departure time occurred earlier (β ± se = −0.085 ± 0.033, *p* = 0.011), while the opposite was true for arrival time (β ± se = 0.107 ± 0.030, *p* < 0.001).

**Figure 3:**
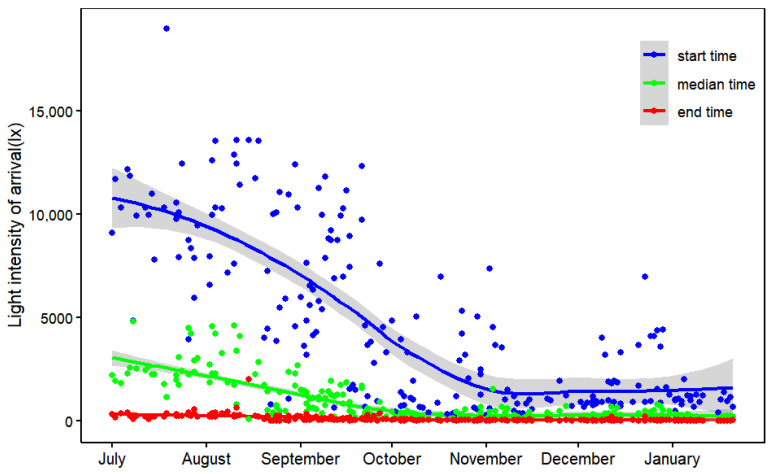
Seasonal changes in light intensity at the start (blue), median (green), and end (red) time of arrival at nocturnal roosts. Lines indicate loess regression, and gray areas indicate standard errors.

Light intensity at the start and median time of arrival showed significantly seasonal variation (Figure 3). Crested Ibises started to return to the roosts under high light intensity before sunset in summer and autumn, but they returned under very low light intensity from November onward (light intensity decreased from 10,187.9 ± 3483.7 lx in July to 787.0 ± 293.8 lx in January; Figure 3). However, the light intensity data at departure time were lowly variable, making it difficult to visualized the relationship between light intensity and departure time. Average light intensity analysis showed that median departure light intensity decreased from 565.7 lx in July to 198.9 lx in November, then increased to 473.7 lx from December to January.

### 3.2. Allocation Between Roosting and Foraging

**Figure 4:**
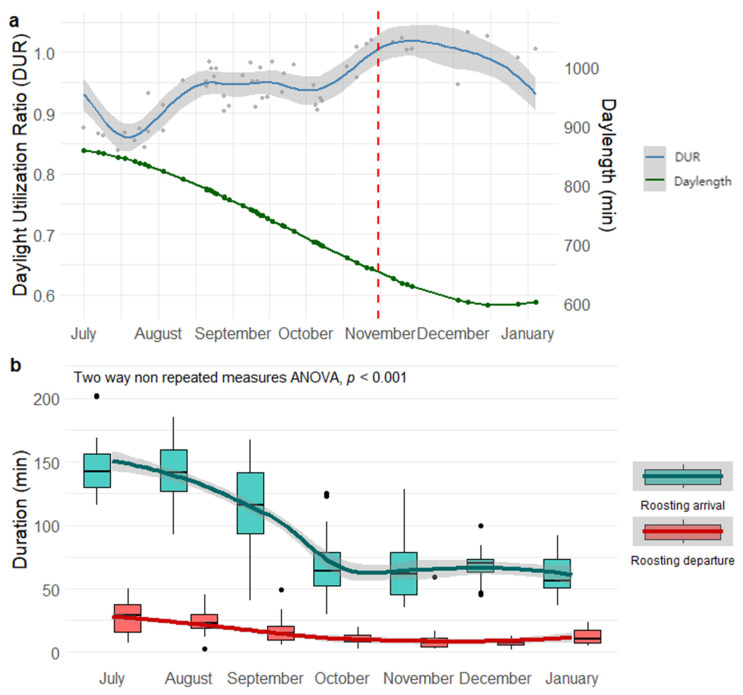
Nonlinear variation in daylight utilization ratio (DUR; *n* = 54) with daylength by the GAM (**a**), and monthly differences in arrival and departure durations of nocturnal roosting in Crested Ibis (**b**). The red dashed line indicates the division between the wandering season and the wintering season. The shaded area represents the 95% confidence interval.

Significant seasonal differences in DUR were detected (χ^2^ = 37.2, df = 6, *p* < 0.001), suggesting that Crested Ibis adjust their daylight activity budgets in response to seasonal environmental pressures. DUR reached a minimum of 0.83 in mid-July and peaked at 1.03 in mid-November. The Generalized Additive Model (GAM) revealed a highly significant non-linear trend in DUR across seasons (edf = 7.85, F = 11.55, *p* < 0.001), explaining 72.7% of the data deviance (Figure 4a). This model quantified the contrasting seasonal patterns: DUR increased with longer daylength during the wintering season but decreased during the wandering season under longer daylength. This divergence implied that daylength alone can’t dictate DUR; instead, it interacted with seasonal ecological constraints (e.g., food availability, thermoregulation needs). On average, the wintering season (0.98 ± 0.01) had a 5.4% higher DUR than the wandering season (0.93 ± 0.00).

Significant monthly differences were observed in arrival and departure durations of nocturnal roosting (*F* = 45.8, *p* < 0.001; Figure 4b), with departure durations (median = 16.5 min, range 2~59 min) much more intensive than arrival durations (median = 93.2 min, range 30~202 min). From July to the following January, departure and arrival durations showed a decreasing trend, suggesting that Crested Ibis populations make more efficient decisions in transitioning between roosting and foraging behaviors to minimize energy consumption as daylength shorten (Figure 4b).

### 3.3. Seasonal Variation in Daily Movement Distance and Activity Level

**Figure 5:**
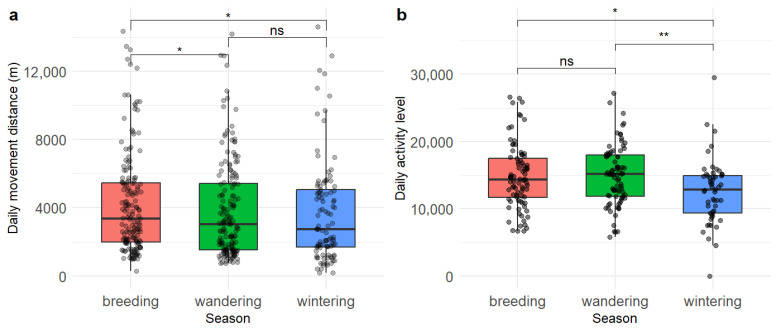
Boxplots of individual daily movement distance (**a**) and daily activity level (**b**) across seasons based on Wilcoxon rank-sum test. Sample sizes for daily movement distance are: breeding season (*n* = 191), wandering season (*n* = 173), wintering season (*n* = 102). Sample sizes for daily activity level are: breeding season (*n* = 84), wandering season (*n* = 85), wintering season (*n* = 53). * *p* < 0.05, ** *p* < 0.01, ns indicates no significance.

The Crested Ibis exhibited pronounced seasonal variations in daily movement distance and activity level. Boxplot analysis revealed that daily movement distance in breeding season was significantly different from that in wandering season (*p* = 0.041) and wintering season (*p* = 0.020), but there was no significant difference between the latter two seasons (Figure 5a). The LMM also confirmed that daily movement distance was significantly shorter during both the wandering (estimate = 912.56, *p* = 0.016) and wintering (estimate = 1068.51, *p* = 0.016) seasons compared to the breeding season, with the wintering season showing an 18.2% reduction (Table 2). No significant difference was found between the wandering and wintering seasons (estimate = 155.95, *p* = 0.916).

The activity level in winter was significantly lower than that in the wandering (*p* = 0.002) and breeding season (*p* = 0.011; Figure 5b). The model indicated that activity level was significantly lower during the wintering season compared to both the breeding season (estimate = 2635.85, *p* < 0.001) and the wandering season (estimate = 2862.86, *p* < 0.001). In contrast, there was no significant difference in activity level between the breeding and wandering seasons (estimate = −227.02, *p* = 0.923; Table 2). This likely reflected an energy allocation trade-off between reproductive effort and winter survival strategies.

## 4. Discussion

In wintering season, Crested Ibises departed nocturnal roosts earlier relative to sunrise and returned later relative to sunset, while the daily movement distance was shorter than that in breeding season. This indicates that in wintering season—with shorter daylight hours—individual movement decreased, but daylight utilization increased.

During the wintering season, most individuals arrived at nocturnal roosts after sunset, significantly later than in July and August, and departed earlier relative to sunrise. This behavioral shift provided approximately 90 min of additional foraging time. Although the Crested Ibis is mainly a tactile forager [36], it relies on a minimum light intensity for flying, locating suitable foraging grounds, and keeping a vigil against natural enemies. Therefore, extending activity into the crepuscular periods at dawn and dusk serves to maximize foraging time. Consistent with this, previous studies also suggested that birds regulate their time allocated to roosting and foraging to cope with seasonal environmental changes, especially in wintering season when food is scarce [11,12]. This finding is powerfully reinforced by studies on the captive population. In captivity, where food is provided sufficiently, ibises reduced foraging time and increased communal roosting timing, particularly under cold conditions [13]. They both strongly suggests that the wild Crested Ibis extended its activity at dawn and dusk primarily as an adaptive response to food scarcity, even at the expense of the thermoregulatory benefits brought by communal roosting. Therefore, our results likely reveal that Crested Ibises strategically use this extra time to compensate for both shortened daylight duration and reduced foraging efficiency during wintering season.

Departure and arrival durations at roost also decreased with the onset of wintering season. This behavioral shift aligns with the general strategy of social birds under food scarcity [9,11]. Moreover, departure durations were significantly shorter than arrival durations, which may be related to decision-making in group coordination and collective behavior [12]. A number of studies have highlighted the importance of food resources in roost selection, noting that foraging areas are usually close to the roosts [37,38]. However, individuals differ in foraging sites and abilities, and successful foragers returning to the roosts earlier [5]. In addition, the large time span of arrival durations may also result from insufficient food resources around the roosts, forcing some roosting individuals to forage in more distant areas.

GPS tracking data showed an 18.2% reduction in wintering daily movement distance compared to the breeding season, and a significantly lower activity level in wintering season compared to breeding and wandering season. This reduction may reflect seasonal changes in food abundance. During food scarcity period—especially wintering—reduced foraging ranges likely represent an adaptive strategy to cope with limited food availability. This behavioral adjustment minimized energy expenditure and enhances survival under resource constraints. Several studies have indicated that the contraction of activity radius in wintering season is closely related to the birds’ dependence on food resources [39,40]. For example, during colder months, some resident birds tend to congregate in limited activity areas to forage, reducing movement needs [41]. This aligns with optimal foraging theory predictions [42,43], whereby animals reduce movement scale to offset energy deficits during resource scarcity while maintaining foraging efficiency [44]. Additionally, reduced movement may compensate for increased thermoregulatory costs, and the positive correlation between movement distance and ambient temperature suggests that cold stress interacts with food restriction. Thus, reduced movement not only helps Crested Ibis survive harsh conditions, but also highlights their ability to adapt to seasonal environmental changes.

Furthermore, shorter daylight hours and lower temperatures reduce the activity intensity of Crested Ibises, leading them to reduce long-distance movement and remain active near fixed feeding grounds [13,45]. Significant seasonal differences in daylight utilization indicate that birds’ behavioral rhythms are highly sensitive to changes in daylight duration [9,46]. By adjusting winter activity times and increasing daytime utilization, they maximize foraging time during cold, food-scarce period while reducing activity and energy expenditure. Higher winter DUR likely reflects intensified foraging efforts to compensate for energy deficits under shorter daylight, while relatively lower DUR in the wandering season may indicate reduced energy demands or behavioral shifts toward communal roosting. The DUR reached a minimum in mid-July, partly due to long daylength and the reduced energy demands after breeding, and a maximum in mid-November, due to short daylength, low temperatures, and increased energy demands for the upcoming breeding season. Therefore, restoring foraging habitats and food resources around nocturnal roosts may play a vital role in reducing winter mortality and promoting the long-term recovery of this endangered species.

## 5. Conclusions

Our findings demonstrate that Crested Ibis extended their outside activity into the periods at dawn and dusk, significantly increased their overall daylight utilization, and concurrently reduced their daily movement distance and activity levels in winter. The combination of these strategies may be explained by the behavioral plasticity of Crested Ibis to cope with the challenging environment in winter. In contrast to studies focusing solely on habitat dynamics, our research highlights the critical role of behavioral plasticity as an ecological adaptation mechanism. To ensure the long-term conservation and population recovery of this species, management efforts should prioritize safeguarding the winter survival needs of the endangered Crested Ibis.

## Figures and Tables

**Table 1:** Information of GPS tracking Crested Ibises.

No.	Age	Birth Year	Sex	Start Tracking Date	Tracking Days	Reason of Tracking Failure
1	Adult	2012	Female	12 August 2014	1786	Out of power
2	Adult	N/A	N/A	10 April 2015	1915	Tag malfunction
3	Juvenile	2015	N/A	24 July 2015	2309	Tag malfunction
4	Juvenile	2016	N/A	9 July 2016	892	Unknown
5	Juvenile	2017	Male	8 June 2017	311	Unknown
6	Juvenile	2017	Male	8 June 2017	340	Unknown
7	Juvenile	2017	Female	8 June 2017	412	Unknown
8	Juvenile	2017	Male	8 June 2017	679	Tag malfunction
9	Juvenile	2017	Female	8 June 2017	796	Unknown
10	Juvenile	2017	Female	8 June 2017	1204	Unknown
11	Juvenile	2018	N/A	17 May 2018	1356	Death
12	Juvenile	2018	N/A	1 July 2018	1012	Death
13	Adult	N/A	N/A	6 April 2021	589	Out of power
14	Adult	N/A	N/A	19 April 2021	443	Death
15	Sub-adult	2020	N/A	30 April 2021	550	Unknown
16	Adult	N/A	N/A	21 July 2021	252	Unknown
17	Juvenile	2021	N/A	21 July 2021	406	Out of power
18	Adult	N/A	N/A	21 July 2021	1029	Unknown
19	Adult	N/A	N/A	16 September 2022	426	Unknown

**Table 2:** Seasonal significance of individual diurnal movement pattern based on post hoc pairwise comparisons.

	Seasons	Estimate	SE	df	t	*p*
Daily movement distance	Breeding vs. Wandering	912.56	328.87	445.38	2.775	0.016 *
	Breeding vs. Wintering	1068.51	384.63	445.58	2.778	0.016 *
	Wandering vs. Wintering	155.95	391.21	445.37	0.399	0.916
Daily activity level	Breeding vs. Wandering	−227.02	596.76	206.37	−0.380	0.923
	Breeding vs. Wintering	2635.85	687.01	207.42	3.837	<0.001 ***
	Wandering vs. Wintering	2862.86	683.11	206.97	4.191	<0.001 ***

* *p* < 0.05, *** *p* < 0.001.

## Data Availability

The original data presented in the study are openly available in FigShare at https://figshare.com/s/2366265922e0c563c7c8 (accessed on 28 August 2025).
